# Stress-induced detwinning and martensite transformation in an austenite Ni–Mn–Ga alloy with martensite cluster under uniaxial loading

**DOI:** 10.1107/S2052252519003208

**Published:** 2019-03-27

**Authors:** Long Hou, Ying Niu, Yanchao Dai, Lansong Ba, Yves Fautrelle, Zongbin Li, Bo Yang, Claude Esling, Xi Li

**Affiliations:** a State Key Laboratory of Advanced Special Steels, Shanghai University, Shanghai 200444, People’s Republic of China; b EPM-Madylam, ENSHMG BP 38402, St Martin d’Heres cedex, France; c Key Laboratory for Anisotropy and Texture of Materials, Northeastern University, Shenyang 110819, People’s Republic of China; d Laboratoire d’Étude des Microstructures et de Mécanique des Matériaux (LEM3), CNRS UMR 7239, Université de Lorraine, Metz 57045, France

**Keywords:** magnetic shape-memory alloys (MSMAs), electron backscatter diffraction (EBSD), detwinning, martensitic transformation

## Abstract

Stress-induced martensitic detwinning and martensitic transformation during step-wise compression in an austenite Ni–Mn–Ga matrix with a martensite cluster under uniaxial loading have been investigated by electron backscatter diffraction, focusing on the crystallographic features of microstructure evolution. The results indicate that detwinning occurs on twins with a high Schmid factor for both intra-plate and inter-plate twins in the hierarchical structure, resulting in a nonmodulated (NM) martensite composed only of favourable variants with [001]_NM_ orientation away from the compression axis. Moreover, the stress-induced martensitic transformation occurs at higher stress levels, undergoing a three-stage transformation from austenite to a twin variant pair and finally to a single variant with increasing compressive stress, and theoretical calculation shows that the corresponding crystallographic configuration is accommodated to the compression stress. The present research not only provides a comprehensive understanding of martensitic variant detwinning and martensitic transformation under compression stress, but also offers important guidelines for the mechanical training process of martensite.

## Abstract 2

A dual-phase Ni–Mn–Ga alloy, composed of an austenite matrix with stably existing martensite embryos, was designed for the experimental and theoretical investigation of the evolution of the crystallographic structure during step-wise stress-induced martensite detwinning and martensite transformation. This research provides an insight into the detwinning mechanisms of a hierarchical twinning structure and crystallographic characterization of a three-stage austenite–martensite transformation.

## 1. Introduction   

Deformation-induced martensitic detwinning and martensitic transformation are fundamental issues in shape-memory alloys (SMAs) and they play important roles in many unique properties, such as giant magnetic field-induced strain (Sozinov *et al.*, 2002 ▸), super-elasticity (Tanaka *et al.*, 2010 ▸) and magnetocaloric and elastocaloric effects (Liu *et al.*, 2012 ▸; Bonnot *et al.*, 2008 ▸). As typical members of ferromagnetic SMAs, Ni–Mn–Ga alloys have recently attracted a great deal of attention owing to their excellent magnetic shape-memory effect (MSME), being conceived as promising sensor and actuator materials (Dunand & Müllner, 2011 ▸; Sozinov *et al.*, 2002 ▸; Chernenko & L’vov, 2008 ▸; Marioni *et al.*, 2005 ▸). Recently, the deformation-related training process of as-grown Ni–Mn–Ga single crystals has been analysed to some extent in several studies (Chulist *et al.*, 2010 ▸; Wang & Khachaturyan, 2006 ▸; Müllner & King, 2010 ▸). In particular, the detwinning evolution of Ni–Mn–Ga thin films under tensile stress has been successfully observed by *in situ* transmission electron microscopy (TEM) (Zárubová *et al.*, 2012 ▸; Ge *et al.*, 2009 ▸). However, the detwinning process and transformation mechanism during a step-wise compression process for martensitic embryos within bulk austenite Ni–Mn–Ga alloys, which is more practical compared with TEM specimen preparation, have rarely been investigated.

In order to obtain the crystallographic features during the evolution of multiple martensitic variant detwinning and of martensitic transformation under mechanical loading, the morphology of a nucleated martensitic cluster was examined, and the crystal orientation relationship (OR) between low-symmetry wedge-like martensite and high-symmetry parent austenite was also determined. We now confine ourselves only to following the crystallographic evolution of a martensite cluster within an austenite Ni–Mn–Ga alloy under uniaxial loading. In this paper, using electron backscatter diffraction (EBSD), the detwinning process of nonmodulated (NM) martensite and an austenite–martensite transformation during step-wise uniaxial compression are analysed in detail. The crystallographic configuration of the inter- and intra-plate boundaries during the detwinning process and the corresponding Schmid factors are investigated. Moreover, the route of the stress-induced martensite transition and its explanation based on crystallographic distortion are discussed. The present research not only provides a comprehensive understanding of martensitic variant detwinning and martensitic transformation under compression stress, but also offers important guidelines for the mechanical training process of martensite.

## 2. Experimental   

Ni–Mn–Ga alloys with the nominal composition of Ni_48_Mn_30_Ga_22_ (at.%) were prepared by arc melting under argon protection, using high-purity Ni (99.99 wt%), Mn (99.9 wt%) and Ga (99.99 wt%) as the raw materials. This alloy was remelted four times and then suction cast into a quartz tube to prepare a cylinder-shaped alloy with a diameter of 3 mm and a length of 150 mm. The cylinder-shaped specimen was then inserted into a high-purity corundum tube with an inner diameter of 3 mm and a length of 200 mm for directional solidification. The samples were prepared by directional solidification at a growth rate of 20 µm s^−1^ through a Bridgman–Stockbarger-type furnace under a transverse magnetic field with an intensity of 0.1 T. The section in the solid zone with stable growth was cut for microstructural characterization and further compression procedures. The sample surfaces were first mechanically polished and then electrolytically polished, with a solution of 20% nitric acid in methanol at room temperature, for the microstructure and orientation observation.

Microstructural and crystallographic characterizations were performed by scanning electron microscopy (SEM/FEI QUANTA 450) equipped with a Hikari high-speed EBSD camera. All EBSD data were analysed using the *OIM* analysis software (EDAX, USA). It should be noted that the NM martensitic plates are composed of alternately distributed fine lamellae with a width of hundreds of nanometres (Cong *et al.*, 2011 ▸), which is smaller than the EBSD resolution by auto-indexing because the interaction area of incident electrons is in the range of several micrometres. As discussed in our previous reports, the crystallographic orientations of microlamellae can be determined by manually indexing the Kikuchi pattern acquired from two neighbouring martensitic variants (Yang *et al.*, 2013 ▸; Li *et al.*, 2013 ▸). The orientation maps in this article were all obtained by auto-collective software, whereas precise information for calculating crystallographic configurations was obtained by manually indexing Kikuchi patterns.

## 3. Results and discussion   

### 3.1. Crystallographic characterization of stress-induced detwinning and martensite transformation by EBSD tracing   

**Figure 1:**
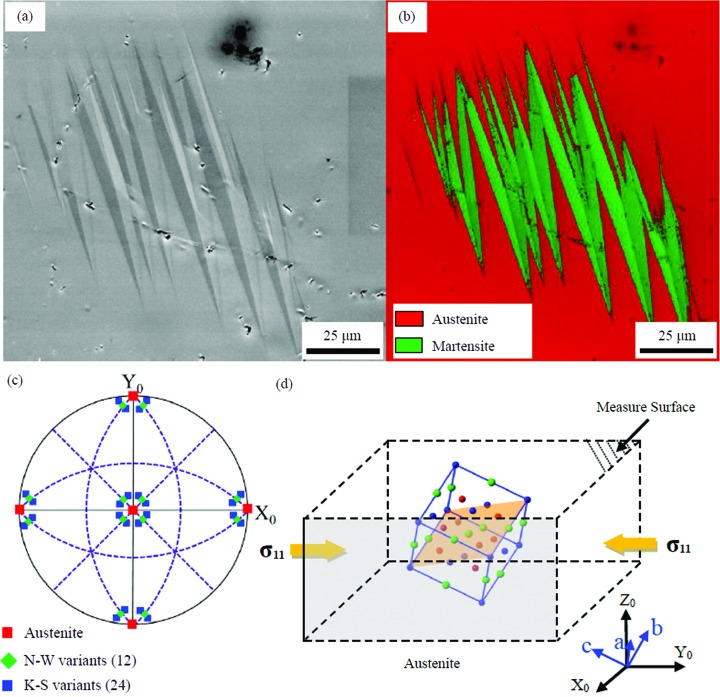
(*a*) The microstructure and (*b*) the corresponding phase map of a martensitic cluster in an austenite matrix. (*c*) An (001) standard stereographic projection showing the austenite and the corresponding produced martensite variants based on N-W and K-S orientation relationships. (*d*) A schematic illustration of *in situ* compression testing with crystal (blue) and sample (black) coordinate systems.

Fig. 1 ▸(*a*) shows the microstructure of the martensitic cluster, *i.e.* some wedge-like variants within the austenite matrix, which can be verified by the EBSD phase determination shown in Fig. 1 ▸(*b*). Fig. 1 ▸(*c*) shows the (001) standard stereographic projection, showing the austenite and the corresponding produced martensite variants based on the Nishiyama–Wassermann (N-W) and Kurdjumov–Sachs (K-S) relationships (Nishiyama, 1934 ▸; Wassermann, 1935 ▸; Kurdjumov & Sachs, 1930 ▸).

**Figure 2:**
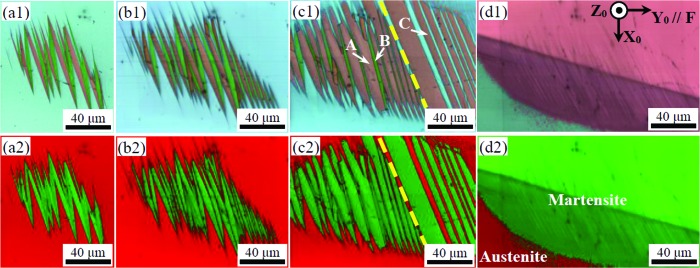
The crystallographic evolution of an NM martensite cluster in an austenite matrix under increasing axial loading. (*a*1)–(*d*1) Orientation images (IPF mode). (*a*2)–(*d*2) Phase maps determined by EBSD. The yellow dashed lines separate the two zones of martensite detwinning and martensite transformation. For easy depiction, in the orientation imaging maps [panel (*c*1)] the red and green plates are denoted Plate A and Plate B, respectively, and the stress-induced martensite plate is denoted Plate C.

Fig. 1 ▸(*d*) depicts a schematic illustration of the *in situ* compression testing used in this work. The corresponding crystallographic evolution under an increasing compression load is shown in Fig. 2 ▸. The evolution under a loading strain can be divided into two regions containing, respectively, stress-induced martensitic detwinning and stress-induced martensitic transformation, and these are separated, in Fig. 2 ▸(*c*), by a yellow dashed line. When the compression stress is small, stress-induced martensitic detwinning dominates [see Fig. 2 ▸(*b*)]. As shown in the orientation imaging maps [Figs. 2 ▸(*b*1)–2 ▸(*d*1)], the red martensitic plate grows while the green martensitic plates shrink and disappear when the loading stress increases further. For clarity, the red and green plates are denoted Plate A and Plate B, respectively, in the present work. When the compression load increases up to a certain value, the martensitic transformation occurs, as shown in Figs. 2 ▸(*c*1) and 2 ▸(*c*2). The emerging martensitic plates are long and parallel, distributed alternately in the austenitic matrix. It should be noted that the emerging martensitic plates (denoted Plate C) in Fig. 2 ▸(*c*1) have the same variant combination as the detwinned martensitic plates (Plate A).

**Figure 3:**
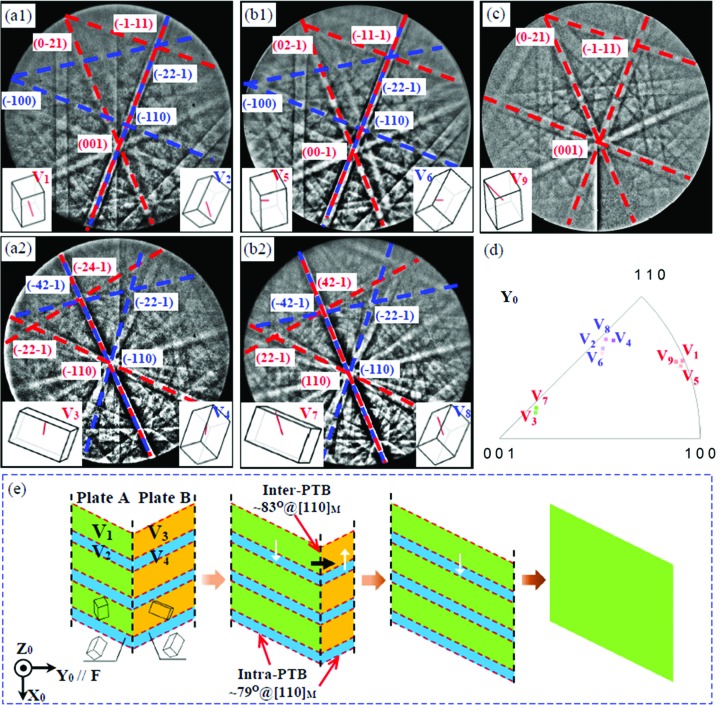
Typical Kikuchi patterns for Plates A and B in the NM martensite cluster embedded in an austenite matrix under increasing axial load. (*a*) The initial stage [shown in Fig. 2 ▸(*a*)], (*b*) the intermediate stage [shown in Figs. 2 ▸(*b*) and 2 ▸(*c*)] and (*c*) the final stage [shown in Fig. 2 ▸(*d*)]. (*d*) The corresponding inverse pole figure along the *Y*_0_ axis (compression direction). (*e*) An illustration of the detwinning process for hierarchically structured non-modulated martensite variants with respect to inter-plate twinning boundaries (Inter-PTBs) and intra-plate twining boundaries (Intra-PTBs).

In order to gain an insight into the crystallographic evolution of martensitic clusters in an austenitic matrix under increasing loading, typical EBSD Kikuchi patterns collected from each compression stage were indexed and analysed. Fig. 3 ▸ shows the typical Kikuchi patterns of detwinned Plate A and Plate B in an NM martensite cluster embedded in an austenite matrix under an increasing axial loading. As mentioned above, the overlaid Kikuchi patterns are composed of major (thick) and minor (thin) martensitic nanolamellae. The martensite plates are composed of two alternately distributed nano-martensitic variants at the initial stage shown in Figs. 3 ▸(*a*1)–3 ▸(*a*2), corresponding to Fig. 2 ▸(*a*). According to the indexed Kikuchi patterns, the schematic orientations of the major and minor martensitic variants are shown in the bottom left and right of each pattern, respectively.

The twinning relationships between the different martensitic variant pairs have been calculated and are shown in the supporting information (Table S1). The inter-plate major–major variant pair and intra-plate major–minor variant pair are compound twinned and the twinning elements are determined as: *K*_1_ = {112}_NM_, *K*_2_ = {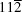}_NM_, η_1_ = 〈
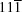
〉_NM_, η_2_ = 〈111〉_NM_, *P* = {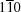} and *s* = 0.353 (*K*_1_ is the twinning plane, *K*_2_ the conjugate twinning plane, η_1_ the twinning direction, η_1_ the conjugate twinning direction, *P* the plane of shear and *s* the magnitude of shear). However, although the shrinkage of the martensite plates is obvious when the compression loading is applied [shown in Fig. 3 ▸(*b*), corresponding to Figs. 2 ▸(*b*) and 2 ▸(*c*)], the detwinning of intra-plate martensitic variants is not obvious.

Finally, when the compression stress increases further, Plate B disappears and the major variants in Plate A are retained.

The Schmid factor is a geometric factor defined as *S* = cos λ cos κ, with λ and κ being the angles between the external uniaxial stress direction and the shear direction and the normal of the twinning plane, respectively. The Schmid factors for the corresponding twinning variant pairs under compression loading shown in Fig. 3 ▸ are listed in Table 1 ▸. The detwinning occurs on the variant pair with a high Schmid factor for both intra-plate and inter-plate twins.

The orientations of nine typical reference variants are shown in an inverse pole figure along *Y*_0_ [the compression axis; Fig. 3 ▸(*d*)]. Briefly, the long axis of NM martensite (*c* axis) is not favoured under compression loading, which explains why the *c* axis of the retained variant after the detwinning process is far away from the compression direction. Combined with the shrinkage of the martensite plates in Fig. 2 ▸, the detwinning of inter-plate martensite variants dominates, and then the detwinning of the intra-plate martensite variant in Plate A occurs. The detwinning process is shown schematically in Fig. 3 ▸(*e*).

**Figure 4:**
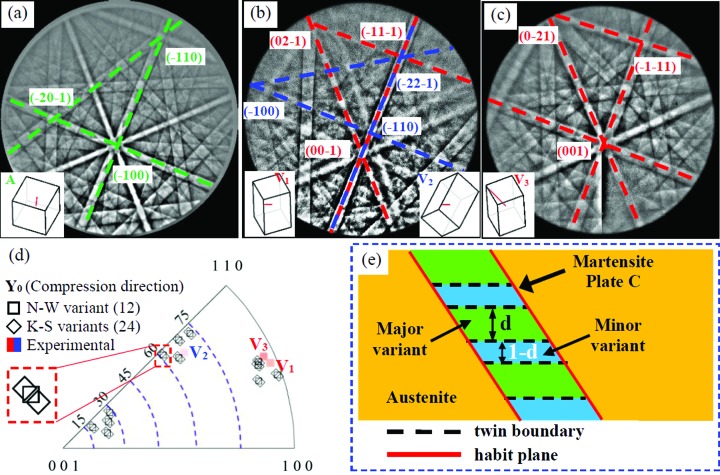
Typical Kikuchi patterns for the austenite–martensite transition under increasing axial load. (*a*) The austenite matrix, (*b*) twinned martensitic variants and (*c*) the detwinned martensitic single variant. (*d*) The corresponding inverse pole figure for martensite variants along the *Y*_0_ axis (compression direction). (*e*) A sketch of the geometry of a twin plate nucleated in an austenite matrix.

Fig. 4 ▸ shows typical Kikuchi patterns for the austenite–martensite transition under an increasing axial load. Fig. 4 ▸(*a*) shows a Kikuchi pattern of the austenite matrix. Fig. 4 ▸(*b*) shows overlaid Kikuchi patterns, which can be verified from a twinned martensite variant pair. When the compression stress is high enough, an obvious Kikuchi pattern from a detwinned single variant can be found [Fig. 4 ▸(*c*)]. The emerging martensitic variant pair are compound twinned too.

The martensitic transformation relationship was also examined by comparing the corresponding pole figures (seen in the supporting information, Fig. S1). During the martensitic transformation, the major variants are in good agreement with the N-W relationship and the minor variants with the K-S relationship. Fig. 4 ▸(*d*) shows the corresponding inverse pole figure for martensite variants along the *Y*_0_ axis (compression direction). It also shows the possible poles calculated theoretically based on the N-W relationship ({111}_A_//{101}_M_, 〈
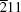
〉_A_//〈
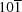
〉_M_) and the K-S relationship ({111}_A_//{101}_M_, 〈
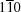
〉_A_//〈
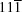
〉_M_). On the one hand, it can be seen that, during the martensitic transformation under compression, the austenite has changed into a twinned martensitic pair instead of a single martensite variant. On the other hand, the stress-induced martensite variants are among the favourably orientated ones. In principle, without stress applied, the probability of variants forming is theoretically identical, but under an external compression stress specific variants can be favoured.

Finally, the stress-induced martensite variant pair should be detwinned into a single variant having the same orientation as the detwinned variant shown in Fig. 3 ▸(*c*). Fig. 4 ▸(*e*) shows a sketch of the geometry of a twin plate, composed of a major variant and a minor variant, nucleated in an austenite matrix, where *d* denotes the ratio of the major variant in a martensite plate, and the corresponding ratio of the minor variant is (1 − *d*).

### 3.2. Strain accommodation in austenite–martensite transformation with respect to the sample coordinate system   

In order to explain the discovered stress-induced martensite transformation behaviour, an analysis is presented based on the self-accommodation characteristics of the produced martensite variant distribution and the corresponding internal stress. Considering the above-verified ORs between the austenite and the produced martensite variant, as mentioned by Zhang *et al.* (2017 ▸), the deformation gradient tensors of 12 theoretical variants under an N-W relationship and 24 theoretical variants under a K-S relationship in the crystal coordinate system were calculated (shown in Tables S2 and S3, respectively, in the supporting information). Here, the lattice parameters are *a*_0_ = 5.840 Å for the austenite phase, and *a* = *b* = 3.862 Å and *c* = 6.557 Å for the produced martensite phase. As mentioned above, the stress-induced martensite plate (Plate C) has the same variant combination as Plate A under a moderate stress load during the martensite transformation [seen in Fig. 2 ▸(*c*)] and is detwinned into the same single variant with increasing stress. Therefore, the deformation gradient tensors of the variant combination (*i.e.* Plate A and Plate B) and the respective single variants (*i.e.* Variants 1 and 2 in Plate A, and Variants 3 and 4 in Plate B) are investigated. All these variants detected experimentally can be found among the calculated variants (Tables S2 and S3 in the supporting information). The major variants (Variant 1 in Plate A and Variant 3 in Plate B) correspond to V_9_ and V_1_ in Table S2. The minor variants (Variant 2 in Plate A and Variant 4 in Plate B) correspond to V_8_ and V_12_ in Table S3.

To estimate the overall deformations related to transformation of the different twinning variant pairs, a mean tensor is defined as follows:
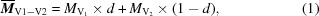
where *d* represents the ratio of the major variant in a martensite plate as mentioned in Fig.4. The thickness ratio between the minor and major variants is 0.597, as determined according to the phenomenological theory of martensitic transformation proposed by Wechsler, Lieberman and Read (WLR theory) (Wechsler *et al.*, 1953 ▸; Wayman, 1994 ▸).Therefore, the macro deformations of Plate A and Plate B in the crystal coordinate system are calculated (shown in Table S4 in the supporting information).

The deformation gradient tensors in the sample coordinate system, 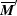, are calculated by 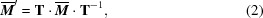
where **T** represents a transformation matrix from the crystal coordinate system to the sample coordinate system based on the Euler angles of the austenite.

Therefore, the deformation gradient tensors 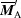 and 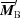 for Plate A and Plate B in the sample coordinate system are as follows: 
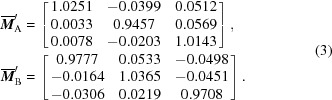



In these deformation matrices, the diagonal element 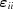 (*i* = 1, 2 and 3) in the tensor represents an elongation (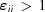) or a contraction (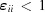) in the *i* direction when the austenite transforms into the martensite. 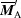, showing a larger contraction (0.9457 for Plate A) along the *Y*_0_ axis, is well accommodated to external stress. When the martensite pairs are detwinned into single variants under increasing compression stress, the deformations 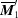, 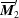, 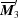 and 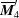 of the four single variants (Variants 1 and 2 in Plate A and Variants 3 and 4 in Plate B) are also calculated (see Table 2 ▸). 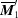, having the largest contraction (0.9348 for the major variant in Plate A) among the four single variants and their variant combinations, can readily adapt to increasing compression stress. This calculation is in good agreement with the above-mentioned experimental results during the stress-induced martensite transformation.

## 4. Conclusions   

In summary, we have presented experimental observation of stress-induced martensitic variant detwinning and stress-induced martensitic transformation during the compression process. Crystallographic analysis of the microstructure evolution of a martensite cluster in an austenite Ni–Mn–Ga alloy under uniaxial loading has been investigated by EBSD. The results indicate that detwinning occurs on twins with a high Schmid factor, resulting in a nonmodulated martensite composed only of favourable variants with [001]_NM_ orientation away from the compression axis. Moreover, the stress-induced martensite transformation occurs at higher stress levels, undergoing a three-stage transformation from austenite to a twin variant pair and finally to a single variant with increasing compressive stress. The findings give an insight into the behaviour of martensitic detwinning and martensitic transformation under compression stress, and also provide important guidelines for the mechanical training process of martensite.

## Supplementary Material

Additional tables and figure. DOI: 10.1107/S2052252519003208/lc5102sup1.pdf

## Figures and Tables

**Table 1:** Schmid factors for the twinning variant pairs under compression loading shown in Fig. 3 ▸

	Intra-plate				Inter-plate	
Host variant	V_1_	V_5_	V_4_	V_8_	V_1_	V_5_
Detwinning variant	V_2_	V_6_	V_3_	V_7_	V_3_	V_7_
Schmid factor	0.0680	0.0741	0.3618	0.3424	0.4263	0.4208

**Table 2:** Deformation gradient tensors ***M*** of four single variants in Plate A (or C) and B presented in the sample coordinate system as illustrated in Fig. 2 ▸; the compressive load is along the *Y*_0_ axis

	Variant type	Deformation gradient tensor, 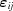		
Plate A (Plate C)	Variant 1 (major)	1.0252	−0.0200	0.1729
		0.0227	0.9348	0.0265
		0.0141	−0.0203	1.0261
	Variant 2 (minor)	1.0248	−0.0732	−0.1527
		−0.0292	0.9639	0.1078
		−0.0027	−0.0204	0.9944
Plate B	Variant 3 (major)	0.9520	0.1380	−0.0404
		−0.0261	1.0860	−0.0441
		0.0076	−0.0442	0.9481
	Variant 4 (minor)	1.0209	−0.0887	−0.0656
		−0.0001	0.9537	−0.0468
		−0.0945	0.1325	1.0086
