# Supplementary material for: Stress-induced detwinning and martensite transformation in an austenite Ni–Mn–Ga alloy with martensite cluster under uniaxial loading
Source: IUCrJ. 2019 Mar 27;6(Pt 3):366–72. doi: 10.1107/S2052252519003208 (PMC6503927; doi:10.1107/S2052252519003208)
Supplement: Supplementary file 1 [file m-06-00366-sup1.pdf]

IUCrJ

Volume 6 (2019)

Supporting information for article:

Stress-induced detwinning and martensite transformation in an austenite Ni–Mn–Ga alloy with martensite cluster under uniaxial loading

Long Hou, Ying Niu, Yanchao Dai, Lansong Ba, Yves Fautrelle, Zongbin Li, Bo Yang, Claude Esling and Xi Li

Table S1 Minimum misorientation angle and the two near-180° misorientation angles, with their corresponding rotation axes, between the intra-plate major and minor twinning variants shown in Fig. 3; for each misorientation angle ω, there is a family of eight equivalent rotations with eight corresponding equivalent axes d, which transform into each other according to the tetragonal rotation symmetry group.

	Variant pairs	Misorientation angle ω (°)	Rotation axis, **d**			
			d_1	d_2	d_3	
Intra-plate nanotwins	V_1/V_2	80.28	0.7040	0.7102	0.0077	0.51° from $\langle 110\rangle_{Tetr}$
		179.68	0.5370	0.5441	0.6446	0.45° from the normal of $(112)_{Tetr}$
		179.43	0.4578	0.4538	0.7645	0.38° from $\langle 111\rangle_{Tetr}$
	V_3/V_4	81.84	0.7112	0.7030	0.0010	0.34° from $\langle 110\rangle_{Tetr}$
		179.56	0.5347	0.5339	0.6550	1.13° from the normal of $(112)_{Tetr}$
		179.93	0.4605	0.4658	0.7556	1.15° from $\langle 111\rangle_{Tetr}$
Inter-plate major-major	V_1/V_3	83.64	0.6997	0.7123	0.0555	3.22° from $\langle 110\rangle_{Tetr}$
		179.32	0.5532	0.5008	0.6657	2.90° from the normal of $(112)_{Tetr}$
		175.76	0.4669	0.4753	0.7458	2.01° from $\langle 111\rangle_{Tetr}$
Inter-plate minor-minor	V_2/V_4	9.74	0.7566	0.0452	0.6523	11.55° from $\langle 301\rangle_{Tetr}$ Or 2.63° from $\langle 201\rangle_{Tetr}$
		175.11	0.7444	0.6660	0.0482	4.21° from the normal of $(110)_{Tetr}$
		179.56	0.0554	0.9964	0.0642	4.86° from $\langle 010\rangle_{Tetr}$
Intra-plate nanotwins	V_5/V_6	82.87	0.7100	0.7042	0.0050	0.37° from $\langle 110\rangle_{Tetr}$
		179.69	0.5325	0.5278	0.6618	1.66° from the normal of $(112)_{Tetr}$
		179.62	0.4660	0.4699	0.7497	1.65° from $\langle 111\rangle_{Tetr}$
	V_7/V_8	81.47	0.7089	0.7053	0.0038	0.26° from $\langle 110\rangle_{Tetr}$
		179.81	0.5375	0.5340	0.6526	0.96° from the normal of $(112)_{Tetr}$
		179.72	0.4602	0.4626	0.7577	0.95° from $\langle 111\rangle_{Tetr}$

Inter-plate major-major	V_5/V_7	84.37	0.7017	0.7101	0.0575	3.31° from $\langle 110\rangle_{Tetr}$
		179.54	0.5513	0.4966	0.6704	3.23° from the normal of $(112)_{Tetr}$
		175.57	0.4772	0.4716	0.7415	2.36° from $\langle 111\rangle_{Tetr}$
Inter-plate minor-minor	V_6/V_8	8.53	0.7114	0.0779	0.6984	15.6° from $\langle 301\rangle_{Tetr}$ Or 6.08° from $\langle 201\rangle_{Tetr}$
		176.18	0.7423	0.6688	0.0415	3.81° from the normal of $(110)_{Tetr}$
		179.34	0.0519	0.9973	0.0529	4.25° from $\langle 010\rangle_{Tetr}$

Table S2 The deformation gradient tensors ***M*** of the theoretically calculated martensite variants under the N-W relationship in the crystal coordinate system.

Variant type	Deformation gradient tensor, ε_{ij}		
V_1	0.9336	-0.0016	-0.0660
	-0.0016	0.9336	-0.0660
	0.0550	0.0550	1.1189
V_2	1.1189	0.0550	0.0550
	-0.0660	0.9336	-0.0016
	-0.0660	-0.0016	0.9336
V_3	1.1189	0.0550	-0.0550
	-0.0660	0.9336	0.0016
	0.0660	0.0016	0.9336
V_4	0.9336	0.0016	-0.0660
	0.0016	0.9336	0.0660
	0.0550	-0.0550	1.1189
V_5	1.1189	-0.0550	-0.0550
	0.0660	0.9336	-0.0016
	0.0660	-0.0016	0.9336
V_6	0.9336	-0.0660	0.0016
	0.0550	1.1189	-0.0550
	0.0016	0.0660	0.9336
V_7	0.9336	0.0016	0.0660
	0.0016	0.9336	-0.0660
	-0.0550	0.0550	1.1189
V_8	0.9336	0.0660	-0.0016
	-0.0550	1.1189	-0.0550
	-0.0016	0.0660	0.9336
V_9	1.1189	-0.0550	0.0550
	0.0660	0.9336	0.0016
	-0.0660	0.0016	0.9336
V_{10}	0.9336	-0.0016	0.0660
	-0.0016	0.9336	0.0660
	-0.0550	-0.0550	1.1189
V_{11}	0.9336	0.0660	0.0016
	-0.0550	1.1189	0.0550

	0.0016	-0.0660	0.9336
V_{12}	0.9336	-0.0660	-0.0016
	0.0550	1.1189	0.0550
	-0.0016	-0.0660	0.9336

Table S3 The deformation gradient tensors ***M*** of the theoretically calculated martensite variants under the K-S relationship in the crystal coordinate system.

Variant type	Deformation gradient tensor, ε_{ij}		
V_1	0.9310	0.0262	-0.1023
	-0.0285	0.9345	-0.0285
	0.0845	0.0262	1.1177
V_2	1.1177	0.0262	0.0845
	-0.0285	0.9345	-0.0285
	-0.1023	0.0262	0.9310
V_3	1.1177	0.0262	-0.0845
	-0.0285	0.9345	0.0285
	0.1023	-0.0262	0.9310
V_4	0.9310	-0.0262	-0.1023
	0.0285	0.9345	0.0285
	0.0845	-0.0262	1.1177
V_5	1.1177	-0.0262	-0.0845
	0.0285	0.9345	-0.0285
	0.1023	0.0262	0.9310
V_6	0.9310	-0.1023	-0.0262
	0.0845	1.1177	-0.0262
	0.0285	0.0285	0.9345
V_7	0.9310	-0.0262	0.1023
	0.0285	0.9345	-0.0285
	-0.0845	0.0262	1.1177
V_8	0.9310	0.1023	0.0262
	-0.0845	1.1177	-0.0262
	-0.0285	0.0285	0.9345
	0.9345	0.0285	-0.0285

V_9	-0.0262	0.9310	0.1023
	0.0262	-0.0845	1.1177
V_{10}	0.9310	0.0262	0.1023
	-0.0285	0.9345	0.0285
	-0.0845	-0.0262	1.1177
V_{11}	0.9345	0.0285	0.0285
	-0.0262	0.9310	-0.1023
	-0.0262	0.0845	1.1177
V_{12}	0.9345	-0.0285	-0.0285
	0.0262	1.1177	0.0845
	0.0262	-0.1023	0.9310
V_{13}	1.1177	0.0845	0.0262
	-0.1023	0.9310	0.0262
	-0.0285	-0.0285	0.9345
V_{14}	0.9345	0.0285	-0.0285
	-0.0262	1.1177	-0.0845
	0.0262	0.1023	0.9310
V_{15}	1.1177	0.0845	-0.0262
	-0.1023	0.9310	-0.0262
	0.0285	0.0285	0.9345
V_{16}	1.1177	-0.0845	0.0262
	0.1023	0.9310	-0.0262
	-0.0285	0.0285	0.9345
V_{17}	0.9345	0.0285	0.0285
	-0.0262	1.1177	0.0845
	-0.0262	-0.1023	0.9310
V_{18}	0.9345	-0.0285	0.0285
	0.0262	1.1177	-0.0845
	-0.0262	0.1023	0.9310
V_{19}	1.1177	-0.0845	-0.0262
	0.1023	0.9310	0.0262
	0.0285	-0.0285	0.9345
V_{20}	0.9345	-0.0285	0.0285
	0.0262	0.9310	0.1023
	-0.0262	-0.0845	1.1177

V_{21}	0.9310	0.1023	-0.0262
	-0.0845	1.1177	0.0262
	0.0285	-0.0285	0.9345
V_{22}	1.1177	-0.0262	0.0845
	0.0285	0.9345	0.0285
	-0.1023	-0.0262	0.9310
V_{23}	0.9345	-0.0285	-0.0285
	0.0262	0.9310	-0.1023
	0.0262	0.0845	1.1177
V_{24}	0.9310	-0.1023	0.0262
	0.0845	1.1177	0.0262
	-0.0285	-0.0285	0.9345

Table S4 The deformation gradient tensors ***M*** of the variant combination in Plate A and Plate B, as illustrated in Fig. 2, in the crystal coordinate system.

Variant pair	Deformation gradient tensor, ε_{ij}		
Plate A	1.0486	0.0038	0.0442
	0.0097	1.0024	-0.0088
	-0.0520	0.0117	0.9339
Plate B	0.9339	-0.0117	-0.0520
	0.0088	1.0024	-0.0097
	0.0442	-0.0038	1.0486

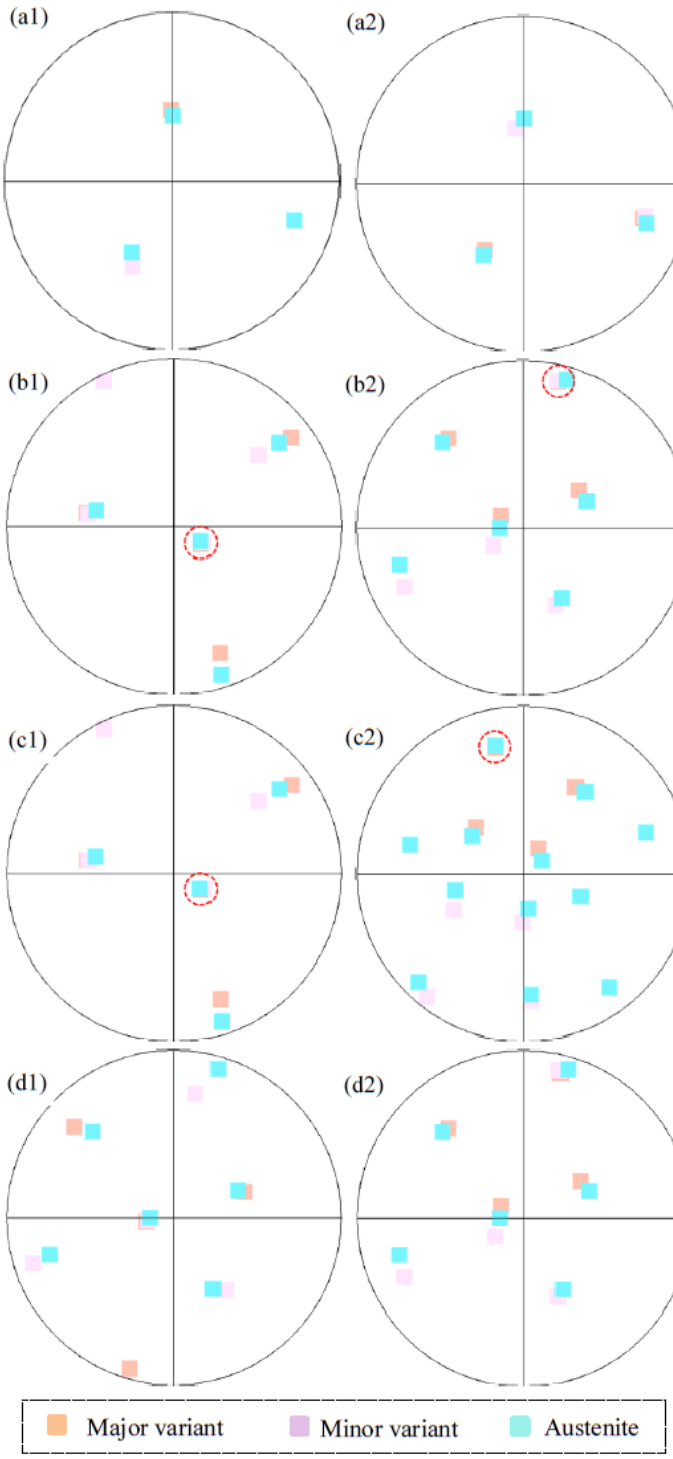

Figure S1. Demonstration of different possible orientation relationships between austenite and martensite, and corresponding pole figures. (a) Bain relation: $(001)_A//(001)_M$, $[100]_A//[1\text{-}10]_M$; (b) K-S relation: $(111)_A//(101)_M$, $[1\text{-}10]_A//[11\text{-}1]_M$; (c) N-W relation: $(111)_A//(101)_M$, $[\text{-}211]_A//[10\text{-}1]_M$; (d) Pitsch relation: $(110)_A//(1\text{-}1\text{-}2)_M$, $[1\text{-}10]_A//[\text{-}11\text{-}1]_M$. The common poles are enclosed with open circles. It indicates that during the martensitic transformation the major variant agrees well with the N-W relationship and the minor variant agrees with the K-S relationship.
